# Supplementary material for: Artificial Cultivation Changes Foliar Endophytic Fungal Community of the Ornamental Plant Lirianthe delavayi
Source: Microorganisms. 2023 Mar 17;11(3):775. doi: 10.3390/microorganisms11030775 (PMC10059682; doi:10.3390/microorganisms11030775)
Supplement: Supplementary file 1 [file microorganisms-11-00775-s001.zip › microorganisms-2251139-supplementary.pdf]

microorganisms

Article

Artificial Cultivation Changes Foliar Endophytic Fungal Community of the Ornamental Plant *Lirianthe delavayi*

Nan-Nan Wu [1,†], Zhao-Ying Zeng [1,2,†], Qin-Bin Xu [1], Han-Bo Zhang [2,*] and Tao Xu [1,*]

[1] School of Ecology and Environmental Science, Yunnan University, Kunming 650106, China
[2] State Key Laboratory for Conservation and Utilization of Bio-Resources in Yunnan, Yunnan University, Kunming 650106, China
* Correspondence: zhhb@ynu.edu.cn (H.-B.Z.); taoxu@ynu.edu.cn (T.X.).
† These authors contributed equally to this work.

Supplementary Material

Supplementary Figures

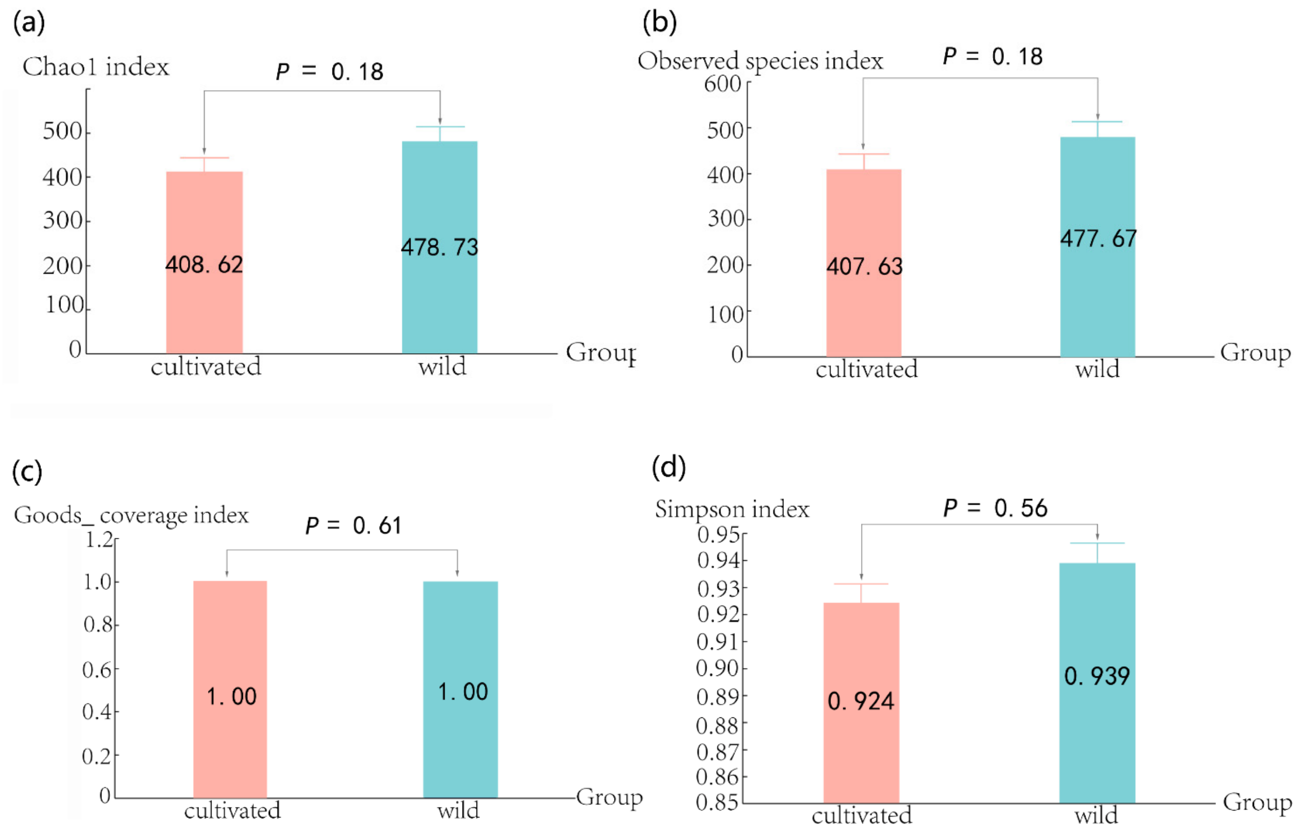

Figure S1. Alpha diversity analysis of endophytic fungi in wild and cultivated *L. delavay* based on the Chao1 index (a), Observed species index (b), Goods_ coverage index (c) and Simpson index (d).

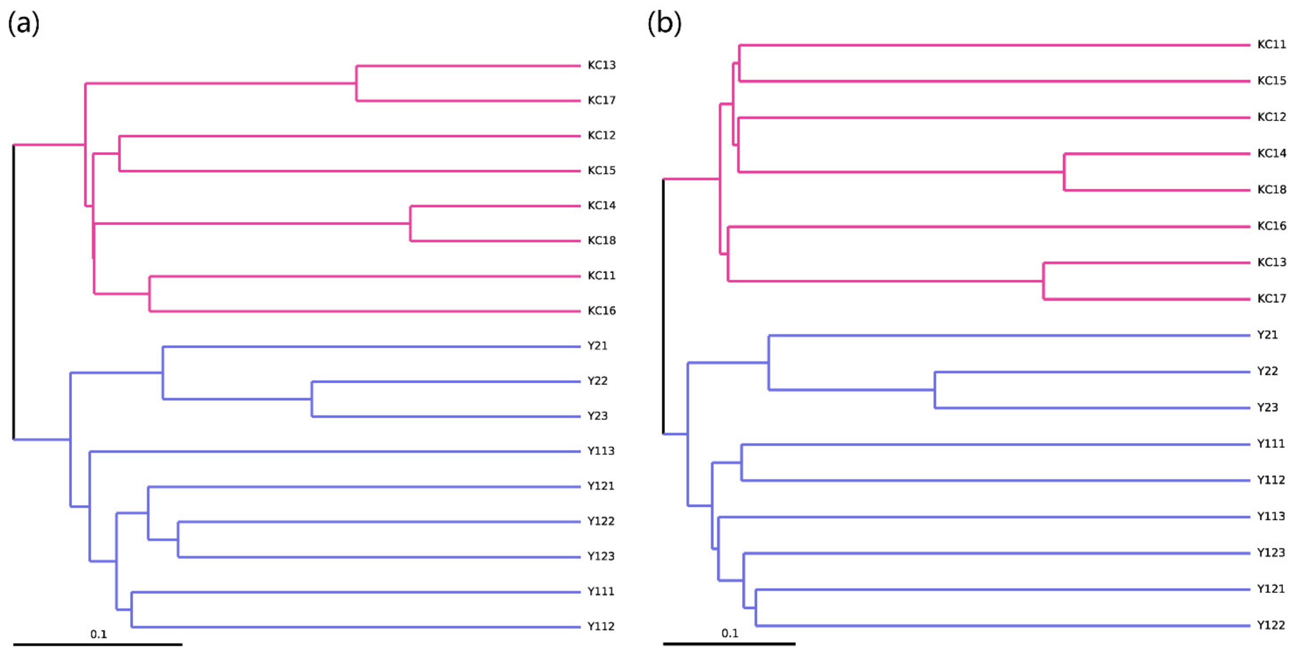

Figure S2. Cluster analysis of endophytic fungi from wild *L. delavayi* and cultivated *L. delavayi* based on the unweighted unifrac distance matrix (a) and jaccard distance matrix (b). Different colors of branches in the figure represent different groups. The cluster tree shows the similarity between samples and the shorter the branch length between samples, the more similar the two samples are. KC represents the leaves of cultivated *L. delavayi*, and Y represents the leaves of wild *L. delavayi*.

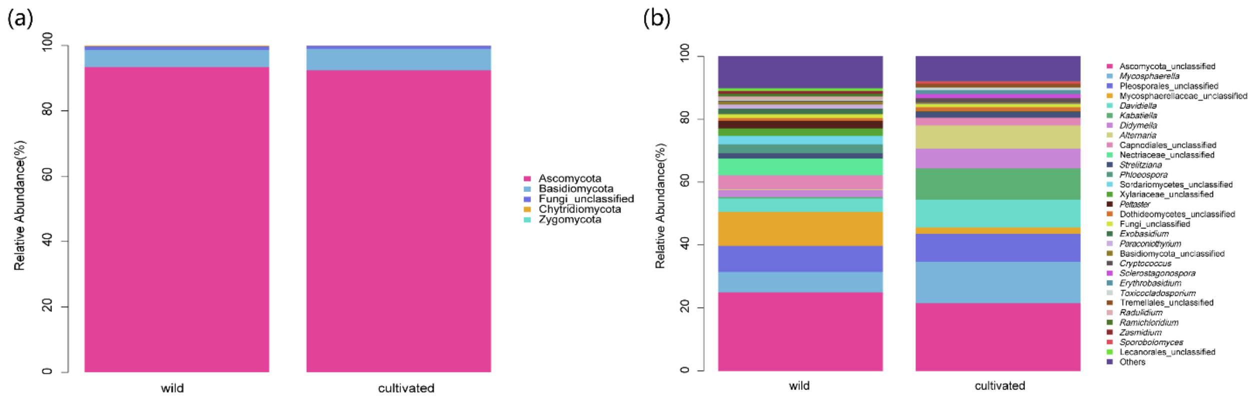

Figure S3. Column stacking diagrams of percentage of endophytic fungi abundance in leaves of wild and cultivated *L. delavayi* at phylum level (a) and genus level (b).

Supplementary Tables

Table S1. Specific information of *L. delavayi* leaves collected from eleven sample plots in Kunming City, Yunnan Province.

Sample No.	Collection location	N°	E°	Altitude /m	Number of collected samples	Plant species distributed in the surrounding environment
Y111, Y112, Y113 (W)	Adong mountain beside S209 County Road, Majie Town, Yiliang County	25°14′12″	103°11′52″	1804	3	*Ageratina adenophora*, *Galinsoga parviflora*, *Pseudognaphalium affine*, *Asplenium trichomanes*, *Pteris cretica* L. var. nervosa, *Artemisia roxburghiana*, *Oxalis corniculate*, *Hypoestes trifloral*, *Hemiphragma heterophyllum*, *Clematis armandii*, *Eremochloa ciliaris*, *Ternstroemia gymnanthera*, *Hedychium spicatum*, *Forsythia viridissima*, *Jasminum grandiflorum*, *Metapanax delavayi*, *Hypericum monogynum*, *Michelia champaca*, *Dioscorea polystachya*
Y121, Y122, Y123 (W)	Adong mountain beside S209 County Road, Majie Town, Yiliang County	25°14′13″	103°11′50″	1794	3	
Y21, Y22, Y23 (W)	Beside S209 County Road, Majie Town, Yiliang County	25°12′35″	103°11′41″	1779	3	
KC11 (C)	Next to Gewu Building, Yunnan University, Chenggong District, Kunming City	24°49′28″	102°51′4″	1975	1	*Celtis kunmingensis* Cheng et Hong, *Euonymus japonicus*, *Duranta erecta*, *Loropetalum chinense* var. rubrum, *Rosmarinus officinalis*, *Ophiopogon japonicus*, *Cuphea hookeriana*, *Hedera nepalensis* var. sinensis, *Hydrangea macrophylla*, *Rose*
KC12 (C)	Next to Gewu Building, Yunnan University, Chenggong District, Kunming City	24°49′28″	102°51′3″	1966	1	
KC13 (C)	Next to Gewu Building, Yunnan University, Chenggong District, Kunming City	24°49′28″	102°51′3″	1971	1	
KC14 (C)	Next to Gewu Building, Yunnan University, Chenggong District, Kunming City	24°49′27″	102°51′3″	1955	1	
KC15 (C)	Next to Gewu Building, Yunnan University, Chenggong District, Kunming City	24°49′27″	102°51′2″	1948	1	
KC16 (C)	Next to Gewu Building, Yunnan University, Chenggong District, Kunming City	24°49′27″	102°51′2″	1945	1	
KC17 (C)	Next to Gewu Building, Yunnan University, Chenggong District, Kunming City	24°49′27″	102°51′2″	1944	1	
KC18 (C)	Next to Gewu Building, Yunnan University, Chenggong District, Kunming City	24°49′27″	102°51′1″	1948	1	
